# Chlorhexidine Gluconate Dressings Reduce Bacterial Colonization Rates in Epidural and Peripheral Regional Catheters

**DOI:** 10.1155/2015/149785

**Published:** 2015-03-23

**Authors:** Klaus Kerwat, Leopold Eberhart, Martina Kerwat, Dominik Hörth, Hinnerk Wulf, Thorsten Steinfeldt, Thomas Wiesmann

**Affiliations:** ^1^Department of Anesthesiology and Intensive Care Medicine, University Hospital Marburg, Baldingerstrasse, 35043 Marburg, Germany; ^2^Institute of Medical Microbiology and Hygiene, Philipps University Marburg, Hans-Meerwein-Strasse 2, 35043 Marburg, Germany; ^3^Department of Trauma, Hand and Reconstructive Surgery, University Hospital Marburg, Baldingerstrasse, 35043 Marburg, Germany

## Abstract

*Introduction*. Bacterial colonization of catheter tips is common in regional anesthesia and is a suspected risk factor for infectious complications. This is the first study evaluating the effect of CHG-impregnated dressings on bacterial colonization of regional anesthesia catheters in a routine clinical setting. *Methods*. In this prospective study, regional anesthesia catheter infection rates were examined in two groups of patients with epidural and peripheral regional catheters. In the first group, regional anesthesia was dressed with a conventional draping. The second group of patients underwent catheter dressing using a CHG-impregnated draping. Removed catheters and the insertion sites were both screened for bacterial colonization. *Results*. A total of 337 catheters from 308 patients were analysed. There was no significant reduction of local infections in either epidural or peripheral regional anesthesia catheters in both CHG and conventional groups. In the conventional group, 21% of the catheter tips and 41% of the insertion sites showed positive culture results. In the CHG-group, however, only 3% of the catheter tips and 8% of the insertion sites were colonised. *Conclusion*. CHG dressings significantly reduce bacterial colonization of the tip and the insertion site of epidural and peripheral regional catheters. However, no reductions in rates of local infections were seen.

## 1. Introduction

In continuous neuraxial and peripheral regional anaesthesia, catheter-related infections are frequent (up to 3%), but mostly localised [1]. However, severe infectious complications may occur such as meningitis or epidural abscess [1]. Despite standardised antiseptic precautions, bacterial colonisation rates of catheters and catheter insertion sites are quite high (up to 60%) [2, 3]. Some authors suggest that a reduction in catheter colonisation rates might lower the incidence of clinical infections, as has been shown for central venous catheters (CVCs). In studies on CVCs, chlorhexidine gluconate (CHG) impregnated-wound dressings reduced microbial catheter colonisation [4, 5] as well as blood-stream infections in high-risk populations [6]. Therefore, the aim of this prospective study was to investigate the effects of a CHG wound dressing on unplanned catheter removal due to suspected local infection (primary outcome parameter), colonisation rates of regional anaesthesia catheters, and catheter insertion sites (secondary outcome parameters) in a routine setting.

## 2. Methods

**Figure 1:**
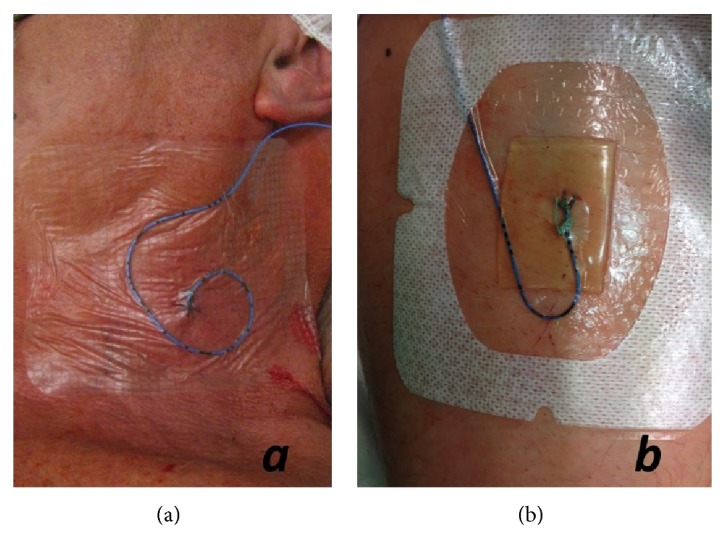
Examples of sterile draping with conventional (a) and CHG dressings (b) in clinical practice.

We prospectively analysed catheter colonisation rates in two groups of patients undergoing operations with neuraxial and peripheral regional anaesthesia catheters from August 2011 to January 2012. In the first group, regional anaesthesia was performed with a conventional dressing using a transparent dressing (IV3000, Smith & Nephew, Hull, Great Britain; see Figure 1(a)). The second group underwent regional anaesthesia after we had modified our local standard operating procedures (SOP) in November 2011. In this group, a dressing with an integrated transparent CHG-impregnated gel pad (Tegaderm CHG, 3M Germany, Neuss, Germany; see Figure 1(b)) was used. Other variables of the local SOP remained unchanged. Regional anaesthesia was performed in the operation room or a designated preparation area taking standard precautions according to local and international practice guidelines [7]. The respective anesthetist (consultant or an experienced resident supervised by a consultant) performing the block was dressed with cap, face mask, sterile gown, and sterile gloves after appropriate hand disinfection according to local guidelines. Skin was prepared using an alcohol-based skin disinfectant (Kodan tinktur forte Spray, Schülke & Mayr, Norderstedt, Germany) following the manufacturer's recommendations. No tunnelling was performed in either group. Peripheral catheters were fixed with a suture before the sterile dressing was applied. Standardized continuous postoperative application of ropivacaine 0.2% was performed using PCA pumps (AmbIt, Teleflex Germany, Kernen, Germany) with standard flow rates (4–6 mL/h) and patient-controlled bolus function (4–6 mL bolus, 30 min lock-out interval).

In order to detect signs of local infections such as erythema and tenderness, daily visual inspection and palpation as well as patient anamnesis were carried out by the acute pain service for each insertion site without removing the dressing according to routine clinical standard.

Signs of regional catheter-associated infections were graded according to the predefined criteria [2, 3, 8, 9] as mild (reddishness, swelling, and painful palpation, ≥2 positive criteria), intermediate (systemic signs of inflammation, e.g., CRP, leukocytosis, fever, and need for antibiotic treatment; ≥2 criteria must be fulfilled), or severe infection (surgical intervention needed).

Catheters were removed according to local practice guidelines when no longer needed or as a result of suspected local infection (mild infection category as described above). Prior to disinfection of the skin and removal of the catheter a sterile swab was used to take a sample from the insertion site and was sent in sterile tubing for microbiological analysis. After antiseptic skin preparation using an alcohol-based skin disinfectant (Kodan tinktur forte Spray, Schülke & Mayr, Norderstedt, Germany), catheter tips were only removed when the skin had dried completely. The tip of the catheter was cut aseptically and transported immediately to the microbiology laboratory.

For microbiological analysis, samples were inoculated on a 5% sheep blood agar plate and in 5 mL thioglycollate broth as described previously [3]. Semiquantitative culture techniques were used as described by Maki et al. [10]. Colony forming units (CFU) were counted and identified using Microflex LT mass spectrometry (Bruker Daltonik GmbH, Fahrenheitsstrasse 4, 28359 Bremen, Germany). Bacterial resistance testing was performed using the Microscan Walkaway 96 (Siemens Healthcare Diagnostics GmbH, 1717 Deerfield Road, Deerfield, IL 60015-0778, USA).

No active randomisation or blinding was performed for this pre- versus postanalysis of a routine clinical practice change. Thus, according to our local ethics committee (2011-08-08, letter from the president of the local ethics committee), no patient consent was required for this quality control survey. Microbiological analysis of regional anesthesia catheter insertion sites or catheter tips is performed in our department from time to time as a part of our local infection surveillance approach for regional anesthesia [11].

Sample size calculation was performed to estimate the needed numbers of catheters. Based on published data, we estimated that the number of unplanned removed regional anesthesia catheters due to suspected infection is 7% in the conventional group and 1% in the CHG group (alpha 0.05 and power 0.80); the required sample size is 164 catheters per group. To cope with potential dropouts, we planned approximately 175 catheters per group to be included in this quality control study.

Data analysis was performed using IBM SPSS (IBM SPSS, release 22, IBM Germany, Ehningen, Germany). Demographic data is presented as mean ± standard deviation.* t*-tests and Chi-square testing were applied when appropriate. A *P* value of 0.05 was deemed significant.

## 3. Results

In total, data of 337 catheters from 308 patients with conventional (*n* = 170) and CHG dressing (*n* = 167) was prospectively collected. Demographic data did not differ significantly between patient groups (see Table 1) except for the indwelling catheter duration in epidural (7.4 days in the conventional group versus 6.0 days in the CHG group) as well as peripheral nerve block catheters (4.6 versus 4.2 days). Of our 308 study patients, 29 received a combination of a femoral and a sciatic nerve block catheter for postoperative pain therapy (15 in the conventional group and 14 in the CHG group) and were subsequently treated as separate cases with regard to individual catheter insertion sites and catheters. No patient showed signs of intermediate or severe infection due to the indwelling regional anesthesia catheter.

With regard to the primary outcome parameter, the rate of catheter removal due to localized infections (mild infection according to the predefined criteria) did not differ significantly between the CHG and the conventional group for epidural catheters (6/60 versus 6/59 catheters, *P* = 0.99). In addition, rates of peripheral catheter removal due to mild infection were not significantly different between both groups (10/96 versus 4/104, *P* = 0.96). For details, see Table 2.

Compared to the conventional draping group, colonisation of the catheter tip and the insertion site was significantly reduced in the CHG group, in both the epidural and the peripheral catheters (Table 2).

In both groups, bacterial cultures from catheter tips and catheter insertion sites showed mainly growth of coagulase-negative* Staphylococcus* spp. (see Table 3).

## 4. Discussion

This is the first clinical study investigating the effects of a CHG dressing for neuraxial and peripheral regional anaesthesia catheters compared to standard catheter dressing with regard to unplanned catheter removal. This endpoint was not significantly different between both groups. However, the CHG dressing resulted in significantly reduced bacterial colonisation of catheter tips and insertion sites in epidural and peripheral catheters compared with conventional dressing.

Local infections following insertion of regional anaesthesia catheters occur in 1–7% of all patients [1, 12–14]. Our results show comparable incidences of local infection signs. Most of these infections are mild and present with erythema and slight tenderness only, without the need for antibiotic therapy or surgical intervention after catheter removal [13]. However, severe complications may occur, such as meningitis or epidural abscess in neuraxial blockades or deep tissue abscess in peripheral nerve blockade [1]. Despite strict aseptic procedures colonisation of the catheter tip and the insertion site is common [3, 15, 16]. Incidences observed for bacterial colonisation of the insertion sites (approximately 41%) and the catheter tips (21%) in our study group with conventional dressings are in line with published data for peripheral [3, 15, 17] and epidural [16] catheters. Different strategies have been tested to reduce colonisation rates in both peripheral and neuraxial catheters such as tunnelling [15] or skin preparation with chlorhexidine gluconate-based antiseptics [18, 19]. Our data supports the potential of CHG to reduce the rates of bacterial colonizations of catheter tips and insertion sites.

Several societies involved in regional anesthesia published recommendations for hygiene in regional anesthesia catheter techniques [7, 8, 11]. Most recommendations are based on assumptions and transfer of evidence-based recommendations of other percutaneous invasive techniques such as central venous catheterization. The updated German guideline for hygiene in regional anesthesia [11] emphasizes the need for further clinical data of hygiene aspects in RA. Our study is the first study investigating the effects of a CHG dressing on infectious complications. As there was no reduction in local infection signs (mild infection as defined above) in a normal population, the clinical benefit of this dressing remains unclear. On the other hand, relevant reductions of bacterial colonization rates showed the potency of the CHG containing dressing. Potentially, studies in high-risk patients for catheter infections (compromised immune system, planned long-term use of regional anesthesia catheters) might reveal clinical benefits besides pure reduction of colonization rates.

Nevertheless, our prospective quality control study has several limitations.

First, we performed this clinical evaluation to evaluate the switch of conventional dressings to CHG dressings in our routine settings. Therefore, the results were obtained as a quality control study but not in a classic randomized, controlled, and double-blinded trial. This is a relevant limitation, as potential bias factors could not have been controlled adequately. We chose the primary outcome parameter “local infection rate” as this is a clinical relevant endpoint. Bacterial colonization rates are potential risk factors for infections but do not necessarily result in relevant infection rates. Further, randomized, controlled, and clinical trials should be performed to achieve higher standards of evidence-based aspects of catheter dressings and their consequences for infectious complications.

Indwelling catheter duration times were longer in both conventional groups compared with the respective CHG group. This might be a potential bias factor for higher colonization rates in the conventional groups per se. There are several possible explanations for these differences. First, our pain service staff might have tend to remove catheters in the CHG group earlier as the insertion sites were not as easy to inspect as in the conventional group. Second, use of the CHG dressing itself might be an independent risk factor for accidental catheter dislodgement with resulting shorter catheter times. This might be underlined by the higher rates of inadvertent catheter removals in the epidural but not the peripheral catheter groups with CHG dressing in our study. Further studies should evaluate this relevant issue for epidural catheters.

Different peripheral insertion sites have different incidences of bacterial colonization. This is important for peripheral as well as neuraxial blockades. As all epidural catheters were thoracic epidurals, incidences of colonization rates should be carefully compared with other studies of both thoracic and lumbar or solely lumbar approaches. Our study sample was too small to evaluate specific peripheral insertion sites and the effects of specific types of dressings. Nevertheless, both groups had comparable distributions of peripheral catheter insertion sites.

In conclusion, this is the first prospective clinical study showing that the use of chlorhexidine gluconate-impregnated dressings results in significantly lower bacterial colonisation rates of catheter insertion sites as well as of catheter tips. On the contrary, the incidences of local infections did not differ between groups. Further randomized, controlled studies should evaluate the impact on clinical infection rates and cost-effectiveness.

## Figures and Tables

**Table 1:** Demographic data of regional anesthesia catheters analysed.

	Conventional	CHG	*P* value^†^
Patients (*n*)	155	153	n/a
Sex (m/f)	70/79	75/71	ns
Age (mean, SD)	56 (17)	56 (18)	ns
Height (cm)	170 (9.3)	171 (9.7)	ns
Weight (kg)	77.2 (17.0)	81.1 (17.2)	ns
BMI	31.2 (6.5)	32.2 (6.3)	ns
Catheters (*n*)	170	167	n/a
Epidural/peripheral	61/109	61/106	n/a
Indwelling epidural catheter duration (days)	7.4 (2.9)	6.0 (2.1)	0.004^†^
Indwelling peripheral catheter duration (days)	4.6 (1.6)	4.2 (1.6)	0.042^†^
Epidural	61	61	n/a
Interscalene	45	49	n/a
Brachial plexus/VIP	4	2	n/a
Femoral	36	33	n/a
Sciatic	24	22	n/a

Conventional: conventional dressing group; CHG: chlorhexidine gluconate group; m: male; f: female; *n*: number; BMI: body mass index; VIP: vertical infraclavicular plexus; ns: not significant; n/a: not applicable; ^†^significant: level of significance *P* < 0.05.

**Table 2:** Local infection rates, catheter colonisation, and catheter removal.

	Conventional (*n* = 170)	CHG (*n* = 167)	*P* value^†^
Local infection as reason for removal of epidural (yes/no)	6/59	6/60	>0.99
Local infection as reason for removal of peripheral catheter (yes/no)	10/96	4/104	0.96
Overall tip colonisation (yes/overall)	35/166	5/161	<0.0001
Overall insertion site colonisation (yes/overall)	70/170	14/162	<0.0001
Epidural tip colonisation (yes/overall)	8/60	0/57	0.0062
Epidural insertion site colonisation (yes/overall)	19/61	5/58	0.0027
Peripheral tip colonisation (yes/overall)	27/106	5/104	<0.0001
Peripheral insertion site colonisation (yes/no)	51/109	9/104	<0.0001
Unplanned removal of epidural catheter (yes/no)	9/59	16/60	0.18
Unplanned removal of peripheral catheter (yes/no)	31/96	32/104	0.88

Conventional: conventional dressing group; CHG: chlorhexidine gluconate group.

^†^significant: level of significance *P* < 0.05.

**Table 3:** Results of bacterial cultures from catheter tips and catheter insertion sites.

Organisms (at tip/insertion site (*n*))	Epidural catheters		Peripheral catheters	
	Conventional (*n* = 61)	CHG (*n* = 61)	Conventional (*n* = 109)	CHG (*n* = 106)
Coagulase-negative *Staphylococcus* spp.	8/18	0/4	19/42	5/8
*Staphylococcus aureus *	0/0	0/0	2/1	0/0
*Enterococcus* spp.	0/0	0/0	3/4	1/1
Enterobacteriaceae	0/0	0/0	4/4	0/1
*Bacillus* spp.	0/1	0/1	3/5	0/1
Others	0/0	0/0	1/1	0/0

Conventional: conventional dressing; CHG: chlorhexidine gluconate group; *n*: number.
